# Quantification of Retrograde Axonal Transport in the Rat Optic Nerve by Fluorogold Spectrometry

**DOI:** 10.1371/journal.pone.0038820

**Published:** 2012-06-18

**Authors:** Christian van Oterendorp, Stavros Sgouris, Michael Bach, Gottfried Martin, Julia Biermann, Jens F. Jordan, Wolf A. Lagrèze

**Affiliations:** University Eye Hospital Freiburg, Freiburg, Germany; Charité University Medicine Berlin, Germany

## Abstract

**Purpose:**

Disturbed axonal transport is an important pathogenic factor in many neurodegenerative diseases, such as glaucoma, an eye disease characterised by progressive atrophy of the optic nerve. Quantification of retrograde axonal transport in the optic nerve usually requires labour intensive histochemical techniques or expensive equipment for in vivo imaging. Here, we report on a robust alternative method using Fluorogold (FG) as tracer, which is spectrometrically quantified in retinal tissue lysate.

**Methods:**

To determine parameters reflecting the relative FG content of a sample FG was dissolved in retinal lysates at different concentrations and spectra were obtained. For validation in vivo FG was injected uni- or bilaterally into the superior colliculus (SC) of Sprague Dawley rats. The retinal lysate was analysed after 3, 5 and 7 days to determine the time course of FG accumulation in the retina (n = 15). In subsequent experiments axona transport was impaired by optic nerve crush (n = 3), laser-induced ocular hypertension (n = 5) or colchicine treatment to the SC (n = 10).

**Results:**

Spectrometry at 370 nm excitation revealed two emission peaks at 430 and 610 nm. We devised a formula to calculate the relative FG content (c_FG_), from the emission spectrum. c_FG_ is proportional to the real FG concentration as it corrects for variations of retinal protein concentration in the lysate. After SC injection, c_FG_ monotonously increases with time (p = 0.002). Optic nerve axonal damage caused a significant decrease of c_FG_ (crush p = 0.029; hypertension p = 0.025; colchicine p = 0.006). Lysates are amenable to subsequent protein analysis.

**Conclusions:**

Spectrometrical FG detection in retinal lysates allows for quantitative assessment of retrograde axonal transport using standard laboratory equipment. It is faster than histochemical techniques and may also complement morphological in vivo analyses.

## Introduction

A characteristic element of neurons is their axon, which, as in retinal ganglion cells (RGCs), can be several orders of magnitude longer than the cell body. To maintain cellular functions in the remote areas of the axon a transport system is required which carries various cargoes, such as proteins and membrane-encapsulated vesicles from the soma to the axon ending (anterogradely) or in the opposite direction (retrogradely).[1]–[3] Axonal transport is different from diffusion as the cargo is actively moved by motor protein ATPases which run along a ‘track’ formed by microtubuli or actin. [4].

In the eye, impairment of retrograde axonal transport is considered an important pathogenic factor in glaucoma [5]–[9], a degenerative disease of retinal ganglion cells (RGCs) which is the second leading cause of irreversible blindness in the world. [10] Besides glaucoma, optic nerve trauma is also characterised by an early breakdown of the axonal transport system. [11].

To assess axonal transport in vivo, tracer substances are introduced into a distinct neuronal area either at the soma or at the axon ending. The amount of tracer that subsequently accumulates at the other end of the neuron is taken as a measure of transport capacity. The detection of the tracer for quantification is most commonly done post mortem on tissue sections using either radioactivity (e.g. I^125^-BDNF) [5], enzymatic activity (mainly horseradish-peroxidase) [12]–[14] or fluorescence. [15] A main limitation of this approach is that significant amounts of tracer may get lost during tissue processing. Furthermore, careful sectioning and densitometric analysis are relatively labour intensive and background noise often largely varies between histological sections.

To allow a more direct measurement of axonal transport different approaches for in vivo detection of tracers have recently been described. They comprise in vivo retinal imaging of fluorescent tracers (Choe TE et al. IOVS 2011;52: ARVO E-Abstract 2448), magnetic resonance imaging with manganese chloride as tracer [11], [16], [17] or direct observation of fluorescently tagged probes by in vivo microscopy. [3] However, these methods require considerable investments and highly skilled personnel, which limits their widespread implementation.

Here, we report on a robust and technically less demanding method to quantify the retrograde axonal transport capacity in the rat optic nerve by using Fluorogold (FG) as tracer substance, which is spectrometrically detected in retinal lysate. FG is known to be taken up by the cell through endocytosis, presumably pinocytosis, There is no passive diffusion through the cell membrane. Packed in endocytotic vesicles it is actively transported in both directions, ante- and retrogradely. [18] FG has been used for decades as the standard dye for retrograde labelling of RGCs. [15] It shows little fading, is non-toxic in standard concentrations and does not leak from axons while transported. [18].

The method consists of three experimental steps:

1. Injection of FG into the superior colliculus.
2. Sacrificing of the animal at a specific timepoint, explantation of the retina and lysis of the retinal tissue following standard protocols for western blotting.
3. Spectrometric analysis of the retinal lysate in a fluorescence microplate reader. The resulting emission data provide a direct measure (c_FG_) for the amount of FG that has accumulated in the retina.

Following the spectrometric measurement, the samples can be further used for specific protein analysis e.g. by western blotting.

## Methods

### Ethics Statement

All animals used in this study were treated in accordance with the ARVO Statement for the Use of Animals in Ophthalmic and Vision Research. The protocols were approved by the Commission on the Use of Animals in Scientific Procedures of the local government (Tierversuchskommission, Regierungspräsidium Freiburg, Germany; permit number: G-10/106).

### Animals

A total of 33 Sprague Dawley rats, weight 275 to 400 g were used for all in vivo and in vitro experiments. The specifications of the rats used for the in vivo experiments were as follows: Time course of FG accumulation in normal retinae: 13 female rats, weight 300–350 g; optic nerve crush: 3 male rats, weight 275–300 g, ocular hypertension: 5 male rats, weight 275–300 g, colchicine injection: 10 male rats, weight 275–300 g.

### Optic nerve crush

Optic nerve crush was performed as previously described. [19] Briefly, rats were anaesthetised with isofluorane. The orbit was opened through an incision at the superior orbital rim and the optic nerve was approached by partially resecting the lacrimal gland. The optic nerve sheath was cut open longitudinally 1–2 mm posterior to the globe, while care was taken not to damage blood vessels. The optic nerve was crushed with blunt forceps for 10 s. Before wound closure the retinal perfusion was checked funduscopically. Animals with severe reduction of the perfusion were excluded.

### Laser-induced Ocular Hypertension Model

Laser treatment to the trabecular meshwork to rise intraocular pressure (IOP) was performed as previously described. [20] Briefly, after intraperitoneal anaesthesia with Ketamine/Xylazine 60 to 70 laser spots from a frequency doubled 532 nm argon laser were directed to whole circumference of the aqueous outflow tract located underneath the periphery of the cornea. Laser setting were 100 µm diameter spot size, 500 mW power and 500 ms duration.

IOP was measured before and at day 1 and 5 after treatment using a TonoLab device (Thiolat, Finland).

### Superior Colliculus Injection of Fluorogold and Colchicine

Animals were deeply anaesthetised with isoflurane. Crystalline Fluorogold (Fluoro-Gold, Fluorochrome, Denver, USA) was resolved as 3% solution in PBS containing 10% DMSO. The skull was exposed and three holes were drilled above the superior colliculus 1 mm lateral to the sagittal and the lambdoidal suture with the central hole being placed at the level of the suture intersection and the other two holes lying 1 mm anterior and posterior to it. A total volume of 3.6 µl FG solution (1.2 µl per hole) was injected using a Hamilton syringe (Hamilton, Bonaduz, Switzerland) mounted on a stereotactic frame (Stoelting, Kiel, Germany). The volume injected into each hole was divided into two equal parts injected at two different levels, 4.2 mm and 4.7 mm below the pia mater. After each injection the needle was left in place for 1 min to avoid reflux of the solution.

For disruption of the microtubular network colchicine (or an equal volume of pure PBS for control injections; Sigma-Aldrich, Munich, Germany) was added to the FG solution. The total injection volume for all three holes was 3.9 µl. The solution contained the same amount of FG as for all other injections plus 0.66 µl of a 30 mg/ml colchicine in PBS solution (or 0.66 µl PBS for control injections).

### Processing of the Retina

Animals were killed with increasing concentration of CO_2_ and the eye was excised from the orbit. To explant the retina, the eye was cut open circularly behind the ciliary body to separate cornea and lens from the posterior portion of the eyeball. The retina was carefully detached from the pigment epithelium and fully separated from the sclera by transection of the proximal optic nerve. The retinal tissue was disrupted with an ultrasound probe (Sonopuls, Bandelin; Berlin, Germany) in 150 µl of standard lysis buffer used for western blotting (RIPA buffer containing standard amounts of protease and phosphatase inhibitors (Complete mini, Roche, Germany; plus 200 µM sodium orthovanadate)). The lysate was kept on ice and used immediately for spectrometry.

### Spectrometry of Retinal Lysate

For spectrometry the samples, each consisting of 140 µl retinal lysate, were transferred to a black 96 well plate (Greiner Cellstar; Frickenhausen, Germany). All measurements were carried out on an Infinite M200 device (Tecan, Crailsheim, Germany). The excitation wavelength was set to 370 nm. Emission was measured between 400 and 700 nm in 10 nm increments. The gain was set constant throughout all measurements.

## Results

We have developed a spectrometric approach to reliably quantify FG in retinal tissue lysates and validated the technique in several in vitro experiments. Subsequently, the spectrometric axonal transport measurement was applied to animals with a healthy optic nerve and to animals with experimentally impaired axonal transport for comparison.

### Spectrometric Quantification of FG in Retinal Lysate

#### The spectrum of Fluorogold in retinal lysate exhibits two peaks at 430 and 610 nm

Before studying FG emission, the excitation wavelength providing maximum emission signal was determined using a 1∶5000 solution of FG in RIPA lysis buffer. Within a range of 300 to 700 nm excitation wavelength (emission detection at 630 nm) a wavelength of 370 nm showed the highest emission (data not shown) and was therefore used throughout all following experiments.

**Figure 1:**
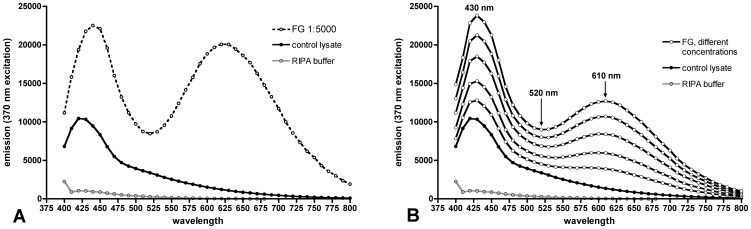
Spectrometry of FG and retinal lysate. **A)** Spectra of pure FG (interrupted line; 1: 5000 dilution) and retinal lysate (black line). The diluent for both was RIPA lysis buffer (grey line). **B)** Spectra of retinal lysate containing different amounts of FG (white dotted lines). Pure retinal lysate without FG (black dots) and RIPA lysis buffer (grey dots) are shown for comparison. With increasing FG concentration the slope of the curve between 520 and 610 nm increases.

The emission spectrum of pure FG in RIPA lysis buffer exhibits two peaks at 440 and 610 nm (Fig. 1A, dashed line). Pure retinal lysate in standard western blot concentration shows a peak of autofluorescence at 425 nm, which slowly decreases with higher wavelengths (Fig. 1A, black line). Testing RIPA lysis buffer alone revealed very low emission between 400 and 500 nm and no significant emission above 500 nm. (Fig. 1A, grey line).

When adding increasing amounts of FG to retinal lysate (Fig. 1B), two peaks emerge at 430 and 610 nm with a trough at 520 nm. (Fig. 1B, white dotted lines) While the peak at 430 nm is a composite result of retinal autofluorescence and FG signal, the second peak at 610 nm is mainly determined by the FG content alone.

#### The relative FG concentration c_FG_ in a sample can be calculated from the peak/trough (610/520 nm) emission

In order to determine the spectrometric parameter that best reflects the FG content in a retinal sample, we conducted in vitro experiments with varying concentrations of retinal protein and FG. When selecting the appropriate parameter from the spectrometric curve, one has to take into account that during excision of the retina from the eyeball, some retinal tissue containing FG is lost, which may vary in different preparations. Consequently, in the analysed sample (which has always a constant volume of sample buffer) the mixture of retinal protein and FG would be more or less diluted. However, given that FG positive RGCs are evenly distributed in the peripheral retina (as demonstrated in supplemental appendix S1 and figure S1) the ratio of FG to retinal protein would always remain constant. For this reason the ideal spectrometric parameter for quantification of the FG content in the retina should reflect the FG/retinal protein ratio rather than the absolute amount of FG in the analysed sample. The 610 nm FG peak emission, however, correlates with the absolute amount of FG and would, therefore, be directly influenced by variations of retinal volume.

**Figure 2:**
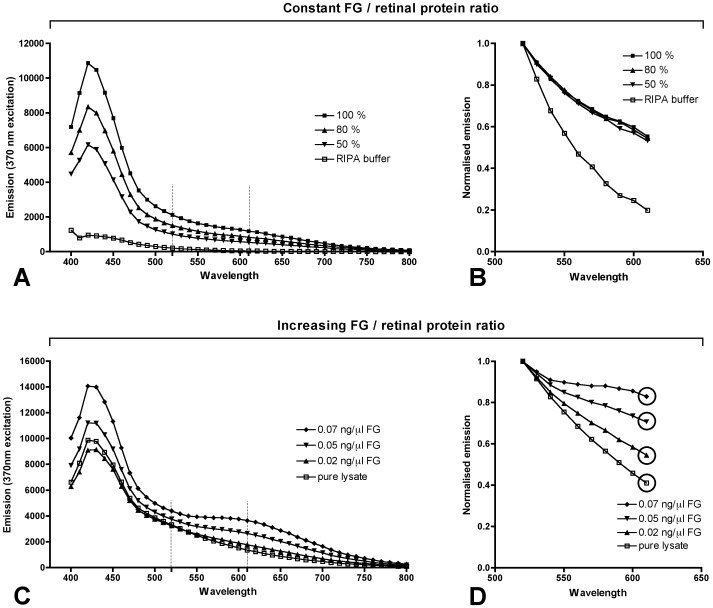
Raw and normalised spectra of retinal lysate from untreated animals with FG added in vitro. **A and B)** Dilution series of retinal lysate containing a constant proportion of FG. 100% is the undiluted sample. 80% and 50% is the content of lysate in the diluted samples. The dilution was done with RIPA lysis buffer. **A)** Raw emission spectra. The dotted vertical lines mark the 520 to 610 nm range that was normalised in B. **B)** Spectra after normalisation to the E_520_ value. The curves of the undiluted and the diluted samples are now congruent, indicating that the normalisation compensates for the effect of different sample dilutions. **C and D)** Retinal lysate with increasing concentrations of FG. **C)** Raw emission spectra. The dotted vertical lines mark the 520 to 610 nm range that was normalised in D. **D)** Spectra after normalisation to the E_520_ value. The end points of the normalised curves are encircled. They increase with higher FG concentration.

The spectrometric curves of retinal lysate with different concentrations of FG in Fig. 1B show that with rising FG/retinal protein ratio the slope of the curve between the 520 nm trough and the 610 nm peak gradually increases. We hypothesised that variations in the dissected retinal volume would result in an up- or downshift of the emission curve by a certain dilution factor. Hence, normalising the emission curves would eliminate the dilution factor and the slope of the normalised curves should reflect the FG/retinal protein ratio. To test this hypothesis we simulated variations in retinal volume by dilution of retinal lysate containing FG with RIPA lysis buffer and compared the resulting raw and normalised emission spectra. To normalise the spectrum between 520 (trough) and 610 nm (second peak) all emission values (E_520 to 610_) were divided by the emission value at 520 nm (E_520_). (Fig. 2 A,B).

The raw spectra indicate decreased signal intensities with increasing sample dilution (Fig. 2A). Normalisation of these emission spectra revealed that the slope of the emission curve (E_520 to 610_) was independent of the sample dilution (Fig. 2B). In contrast, variations of the FG/retinal tissue ratio (Fig. 2C) resulted in different slopes of the normalised emission curve (Fig. 2D). Thus, the curve endpoint (encircled in Fig. 2D), used as a surrogate for the slope, reflects the FG/retinal protein ratio.

To quantify the difference in FG content between experimental samples the fact that the normalised emission (e_n_) for lysate containing no FG (e_n_^0^) is not zero has to be taken into account (see Fig. 2D). The e_n_ of a sample containing FG (e_n_^x^) adds to this baseline value. Thus, to calculate the relative FG content (c_FG_) of a sample the e_n_^x^ value needs to be normalised to e_n_^0^. This results in:
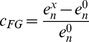


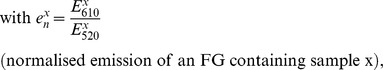


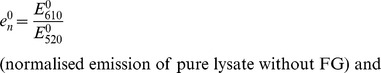


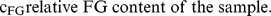



Consequently, besides the groups of experimental and control retinae which provide the e_n_^x^ data a small group of normal retinae without FG from the same strain, sex and age of rats is necessary in order to obtain the background e_n_^0^ values. In our experiments the group size of these normal retina samples was n = 3 and 4. The mean of the e_n_^0^ values was used to calculate the c_FG_ of each experimental sample. Once e_n_^0^ is determined it can be used for all samples as long as animal strain, sex and age remain unchanged.

Supplemental data (see appendix S2 and figure S2) further characterise the quantitative relation of c_FG_ and the real FG content.

### Spectrometric Analysis of FG Retrograde Axonal Transport in the Rat Optic Nerve

#### Time course of FG accumulation in the normal retina

**Figure 3:**
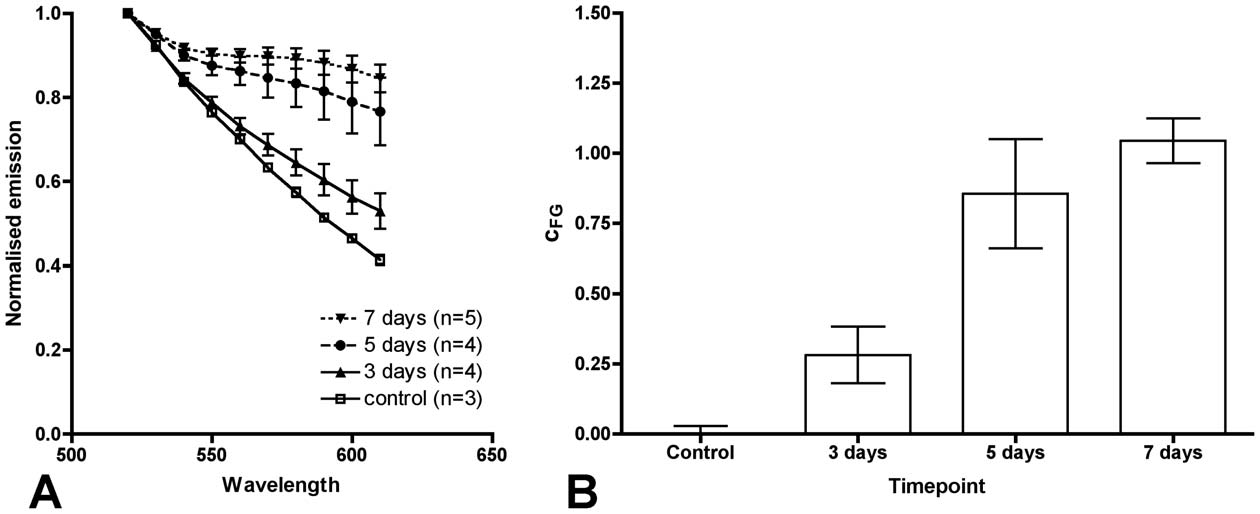
FG accumulation in the retina by retrograde transport over time. Normalised emission curves **(A)** and relative FG content in the lysate (c_FG_; **B**) in control retinal lysate and at three different timepoints after FG injection. The data points and bars are the mean and SEM for each group. The group size is given in panel A. The difference between control and days five and seven are statistically significant (p<0.01 and 0.001, respectively).

We determined the time course of FG accumulation in the retina by FG injection into the right superior colliculus and measurement of the spectrometric signal in the retinal lysate of the contralateral eye after three, five and seven days. Fig. 3 shows the gradual increase of the normalised curve slope and the c_FG_ value with time. The difference between the control eye group and all three timepoints was statistically significant (p<0.0002; one-way ANOVA). In post hoc testing at each individual timepoint versus the control eyes, the difference between the control eyes and day five and seven was significant (day three: p>0.05; day five: p<0.01; day seven: p<0.001; Bonferroni's multiple comparison test).

As the difference between control and day three was not statistically different in the post hoc analysis we chose day five as the timepoint appearing most appropriate to detect differences in FG transport in the following in vivo experiments.

#### Spectrometric quantification of axonal transport impairment

To assess whether the method is capable of detecting axonal transport impairment, we conducted three independent in vivo experiments. Optic nerve damage was induced by either: 1. optic nerve crush for an acute severe axonal injury, 2. ocular hypertension for a more mild axonal damage or 3. colchicine injection in the superior colliculus for pharmacological disruption of the microtubuli network in the optic nerve axons.

For optic nerve crush (n = 3) or laser treatment to induce ocular hypertension (n = 5) FG was injected into both superior colliculi and the damaging treatment was performed unilaterally during the same anaesthesia. In the laser treated eyes the intraocular pressure at day one was 34.8±5.3 mmHg (mean and SEM) higher than in the contralateral control eye. As known for this laser model, the pressure had returned to almost normal level at day five (1.2±3.3 mmHg above control).

For microtubular disruption, colchicine (or PBS for control) was injected together with FG into the right superior colliculus (n = 5 for treatment and control group). The spectrometric measurements were done at the left eye as the contralateral eye is predominantly affected in albino animals due to crossing of more than 90% of the axons.

**Figure 4:**
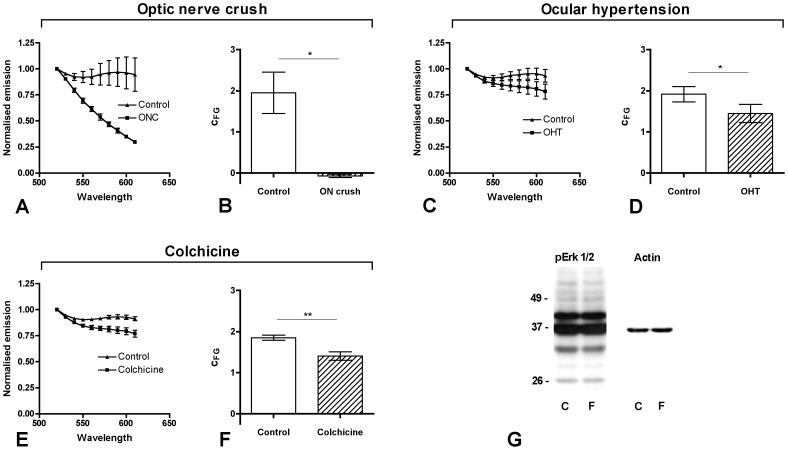
FG accumulation in the retina after different types of optic nerve injury. **A-F)** Normalised emission curves (A, C, E) and c_FG_ (B, D, F) five days after optic nerve injury. Data points and error bars are mean and SEM. **A, B)** Optic nerve crush, n = 3, p = 0.029. **C, D)** Laser induced ocular hypertension, n = 5, p = 0.025. **E, F)** Colchicine injection to the superior colliculus, n = 5 for treatment and control group, p = 0.006). **G)** Western blot for phospho-Erk1/2 from control retinal lysate after superior colliculus injection of PBS (lane “C”) and from retinal lysate used for FG spectrometry (lane “F”). Both lanes show a similar band pattern indicating that despite the FG labelling and spectrometric measurements the retinal lysate can be further used for western blots.

In all cases the c_FG_ value was determined from retinal lysate five days after treatment. The e_n_^0^ used for all calculations was obtained by averaging the measurements of four retinae without FG (e_n_^0^ = 0.324±0.014). All three modes of damage showed significantly less FG in the retinal lysates of treated eyes compared to untreated control eyes (Fig. 4 A-F; c_FG_ mean ± SEM for untreated and treated eye; one-tailed t-test: optic nerve crush: 1.85±0.5 and −0.005±0.004, p = 0.029; ocular hypertension: 1.80±0.19 and 1.53±0.22, p = 0.025; colchicine: 1.80±0.06 and 1.30±0.10, p = 0.006). In the optic nerve crush group c_FG_ was reduced to levels of FG-free controls (Fig. 4 A,B). In the ocular hypertension model with a less severe optic nerve damage, a smaller reduction in FG levels was detected. In the colchicine group, a moderate but highly significant drop in axonal transport capacity was detected (Fig. 4).

After spectrometric measurement the retinal lysate can be further used for western blotting. The FG itself as well as the spectrometry procedure do not seem to cause any interference with the subsequent western blot (Fig. 4G). When probing for phospho-Erk1/2 there was no indication for increased protein degradation or dephosphorylation.

## Discussion

We have developed a method to quantify the retrograde axonal transport capacity in the rat optic nerve by fluorogold (FG) superior colliculus injection and subsequent spectrometric detection of FG in retinal lysate. The relative FG concentration in the tissue lysate, c_FG_, is calculated from the spectrometric emission measured at 610 and 520 nm in the experimental sample and a FG-free reference.

A significant increase of FG signal above baseline was detected from day five after superior colliculus injection. Therefore, this timepoint was chosen in transport interruption studies. Choosing day seven might have resulted in a slightly stronger signal with better differentiation of different grades of transport impairment. However, we found choosing the earliest significant timepoint more sensible because the timecourse of FG accumulation shows a steep rise from day three to five and levels off after day five. This suggests saturation of the neurons and increasing anterograde transport of FG from the soma back to the axon ending at later time points. Also, the FG concentration at the superior colliculus slowly decreases with time as a consequence of diffusion into larger brain areas and drainage to the blood vessels. Therefore, choosing the most dynamic phase of FG accumulation in the retina provides the highest chance to detect a transport impairment in neurodegeneration. For rapidly developing neurodegeneration, even the five day observation time may be too long. But for more slowly progressing disease models like chronic glaucoma or generalised CNS diseases it appears adequate. Even if some RGCs die and release their FG during the observation period, the tracer would still appear in the lysate and be available for detection.

By normalisation of the raw spectra our method compensates for variations in the retinal volume retrieved (Fig. 2). This eliminates the need for a separate method to acquire the sample protein concentration. The normalised emission curve of diluted samples was similar to undiluted samples up to a dilution of 50% (Fig. 2 A,B). This would control for a much higher variability of protein concentration than observed in typical experiments. In a study measuring the weight of nine consecutive retinae dissected in our laboratory we determined a variation coefficient of 11%.

A source of error when using the spectrometric method would be the comparison of groups injected with different preparations of FG working dilutions. Variations in pipetting as well as fading of fluorescence over time may lead to erroneous results. Therefore, each experimental group of animals needs an individual control group injected with the same FG solution, preferably at the same day.

The primary goal in developing the method was to measure axonal transport as a functional parameter. It was not designed to replace RGC counts for neurodegeneration or neuroprotection studies. Although the number of RGCs and the amount of FG in the retina somehow correlate, spectrometry does not distinguish between FG in viable RGCs and FG released from dead RGCs. However, by carefully choosing the timepoint of FG injection and sampling, the spectrometric measurement may provide additional valuable information about RGC survival. For example, in a slowly progressing neurodegenerative disease FG injections at different timepoints each followed by spectrometry five days later would result in a value that is not only dependent on the number of physically present RGCs (like RGC counts) but would also reflect the RGC’s ability to carry out axonal transport. This approach, however, requires careful validation which was beyond the scope of the present work.

Besides the much shorter data acquisition time another advantage of FG spectrometry over histological detection methods is that the tissue lysates can be used for further analyses. The FG present (molecular weight approx. 500 Daltons) does not interfere with SDS-Gel electrophoresis and wet-blotting procedures as demonstrated in Fig. 4G.

Given that axonal transport impairment is considered a key pathogenic factor in a variety of neurodegenerative diseases such as amyotrophic lateral sclerosis, [21] X-linked Charcot-Marie Tooth neuropathy [22] and hereditary spastic paraplegias [23] FG spectrometry could be applied to any nerve and any direction of transport. In cases where cargo binding is affected, such as in Parkinson’s and Alzheimer’s disease [24], different cargo vesicles may be differentially affected. Thus, the degree of transport impairment of FG carrying vesicles may be different from pathogenically relevant vesicles, for example those carrying neurotrophic factors. [25] In all other cases where the disease primarily affects the movement of the motor proteins (dynein or kinesin, e.g. Huntigton’s disease) or the tubular ‘railroad’ (microtubuli) we consider a similar impact on the different types of endocytotic vesicles.

If both FG injection area and target area are located in the brain they need to be anatomically well separated to avoid tracer spillover by interstitial diffusion. The fact that c_FG_ corrects for variations in tissue protein concentration is particularly helpful when working with neuronal projections to the brain, as tissue volumes dissected from a particular brain area often vary largely. However, this does not exclude sampling error. If a significant amount of the dissected tissue is outside the neuron projection zone the c_FG_ will be falsely low.

In contrast to optical in vivo imaging systems which are increasingly used in eye research, FG spectrometry uses ex vivo tissue (retinal lysate) which requires a higher number of animals when investigating different timepoints. Information on spatial differences in the retinal transport between individual RGCs or areas of the retina are lost. However, using a highly sensitive, wavelength-specific and well calibrated detection system, which by its measurement principle excludes a large number of variables occurring in optical imaging in vivo, the spectrometric method is particularly suitable for quantification purposes. This is also reflected by the very low inter-measurement variability of the in vitro measurements shown in shown in supplemental figure S2. Our data suggest, that FG spectrometry may serve as a valuable addition to in vivo imaging. Furthermore, FG spectrometry provides a robust and low-cost tool to study axonal transport in the optic nerve as a stand-alone system if more elaborate and expensive techniques are not available.

## Supporting Information

Appendix S1 **Assessment of RGC density changes in the outer periphery of the retina.**
(DOC)

Appendix S2 **Calculation of the relative FG content.**
(DOC)

Figure S1 **Quantification of RGC density changes in the outer periphery of the retina.** **A)** Schematic of a flatmounted retina and the counting frame (dashed line), which is divided into a central and peripheral sub-frame. The size of the counting frame was enlarged for better visibility. **B)** Box plot of the peripheral to central subframe RGC density (p/c ratio) of 4 retinae. The dashed line indicates a p/c ratio of 1 (no difference between central and peripheral sub-frame). There is no statistically significant difference to the value 1 (p = 0.56) indicating that RGC density does not change as a function of eccentricity in the outer periphery. **C)** Representative image of FG labelled RGCs. Image size equals counting frame size. The horizontal white line indicates the boundary of the sub-frames. Top peripheral part, bottom central part.
(TIF)

Figure S2 **Relationship between relative and absolute FG content.** c_FG_ plotted against r_FG_ of retinal lysate with known amounts of FG added. The data points are the mean and SEM of n = 3 samples. The interrupted line is the linear regression of the data points. The plot demonstrates that the experimentally determined c_FG_ value is proportional to the real FG concentration r_FG_.
(TIF)
