# Supplementary material for: Quantification of Retrograde Axonal Transport in the Rat Optic Nerve by Fluorogold Spectrometry
Source: PLoS One. 2012 Jun 18;7(6):e38820. doi: 10.1371/journal.pone.0038820 (PMC3377715; doi:10.1371/journal.pone.0038820)
Supplement: Appendix S1 — Assessment of RGC density changes in the outer periphery of the retina. (DOC) [file pone.0038820.s001.doc]

**Appendix S1. Assessment of RGC density changes in the outer periphery of the retina**

*Definition of the relevant retinal area*

The retina is connected with the sclera at the equator of the eyeball. By cutting away this connection, variable amounts of the outmost peripheral retina will be lost. As stated in the discussion, the coefficient of variation of the retinal tissue obtained is 11% in our laboratory. We therefore assumed that in the worst case up to 11% of the peripheral retina may be lost. As known the RGC density is highest in the central retina and decreases towards the periphery (Lasseck et al. 2007). However, for our model to reliably compensate for the variable tissue loss the RGC density in the outmost 11% peripheral retinal area needs to be constant.

The estimated size of the area of variability was deduced as follows. The radius of a most peripherally dissected retina is approximately 4.05 mm. By geometric calculation the radius segment of the 11% outer area is 0.284 mm. In microscopy images taken with 200x magnification this equals 947 pixels. For convenience, the region of interest was set to cover a radial segment of 1000 pixels.

*FG labelling and measurement of RGC density*

Four retinae with FG labelled RGCs were flatmounted. From each retina six images of the outmost periphery were taken with a fluorescence microscope at 200x magnification (Fig. S2 c). A counting frame of 1300 pixels width (parallel to the retinal border) and 1000 pixels height was placed in each image (Fig. S2 a). The orientation of each image was such that the upper part faced towards the retinal boundary and the lower part towards the centre. The counting frame was equally divided into an upper and a lower sub-frame of 1300 x 500 pixels (w/h), the upper covering the more peripheral half, the lower the more central half of the original counting frame (Fig. S2 a and c). All FG labelled RGCs were then counted in each sub-frame. As a first approximation to the question whether the RGC density further decreases in the very periphery of the retina, the RGC counts between the peripheral and the central sub-frame were compared by calculating the peripheral / central ratio (p/c ratio). For each retina the p/c ratio of six counting frames was averaged. The p/c ratio of the four retinae was compared to the value 1 (no difference between peripheral and central sub-frame) using a Wilcoxon signed rank test (Fig. S2 b). The mean p/c ratio ± standard deviation was 1.02 ± 0.09. There was no statistically significant difference to the value 1 (p = 0.56, two tailed). Thus, we can state that in the outer periphery of the retina. which is relevant for our model, the RGC density is sufficiently constant as a function of eccentricity.

**Reference**

Lasseck J, Schroer U, Koenig S, Thanos S (2007) Regeneration of retinal ganglion cell axons in organ culture is increased in rats with hereditary buphthalmos. Exp Eye Res 85: 90-104.
