# Supplementary material for: Quantification of Retrograde Axonal Transport in the Rat Optic Nerve by Fluorogold Spectrometry
Source: PLoS One. 2012 Jun 18;7(6):e38820. doi: 10.1371/journal.pone.0038820 (PMC3377715; doi:10.1371/journal.pone.0038820)
Supplement: Appendix S2 — Calculation of the relative FG content. (DOC) [file pone.0038820.s002.doc]

**Appendix S2. Calculation of the relative FG content**

The first step of data processing is normalisation of the raw spectrometric data which compensates for the variation of the emission signal due to variable retinal volumes dissected from the eyeball (Fig. 2 a, b). Normalisation to the E_520_ (trough) value assumes that the changes of this value as a function of FG content is relatively small compared to the end point of the normalisation curve at 610 nm (second peak). The validity of this assumption will be addressed later.

From the spectra of retinal lysate with increasing amounts of FG added in vitro (Fig. 2 c, d) we concluded that the end point values of the normalised emission curves increase in a monotonic fashion with increasing FG concentration. The end point values of the normalised curve are termed e_n_. They are calculated from the emission at 610 nm by normalising with the emission at 520 nm:

(1)

The e_n_ value of a sample containing FG is determined by the real FG content r_FG_ and the baseline emission signal derived from the autofluorescence of the retinal proteins e_n_^0^ (pure lysate curve in Fig. 2d). As we did not calibrate our system to real FG concentrations we introduce the relative FG content c_FG_, which is directly proportional to r_FG_.

The equation to calculate the c_FG_ of a given sample includes two assumptions:

1. Emission due to FG in the retinal lysate adds linearly to the e_n_ value. This was deduced from the normalised emission curves in Fig. 2d.

2. The e_n_^x^ value of a given FG sample needs to be normalised to the e_n_^0^ value because the baseline autofluorescence of the retinal proteins (e_n_^0^) changes with strain, age and sex of the rats used.

Consequently, we calculated the relative FG content as:

(2)

or with e_n_ replaced by the raw emission values:

(3)

The proportionality of c_FG_ to the real FG content (r_FG_) is demonstrated by the following experiment:

If c_FG_ is proportional to r_FG_, then in two different samples (a and b) with a known FG content: . Thus, if c_FG_ is plotted against r_FG_, the resulting data points would be on a straight line.

For experimental confirmation of these assumptions three retinal lysates were subjected to spectrometry and FG was added stepwise to the following final concentrations: 0.019, 0.047 and 0.072 ng/μl FG. The raw emission and normalised data of one sample was used for Fig. 2 c and d. The FG concentrations added in vitro are in the same order of magnitude as the retinal FG concentrations in our in vivo experiments.

As an example the data for one sample was calculated as follows:

The raw emission at 520 and 610 nm was 3274 and 1372, respectively, for the pure lysate without FG and 4478 and 3801 for the 0.072 ng/μl FG concentration.

Thus, following equation (1), e_n_^0^ and e_n_^0.07^ were calculated:

and

Inserting this into equation (2) results in:

and

The c_FG_ of all three samples plotted against the r_FG_ for the FG concentrations 0.019, 0.047, 0.072 ng/μl and pure lysate is shown in Fig. S1. There is a very high linear relation between all groups, which demonstrates that the c_FG_ value calculated from the spectrometric data is indeed proportional to the real FG content.
