# Supplementary figures and images for: Quantification of Retrograde Axonal Transport in the Rat Optic Nerve by Fluorogold Spectrometry
Source: PLoS One. 2012 Jun 18;7(6):e38820. doi: 10.1371/journal.pone.0038820 (PMC3377715; doi:10.1371/journal.pone.0038820)

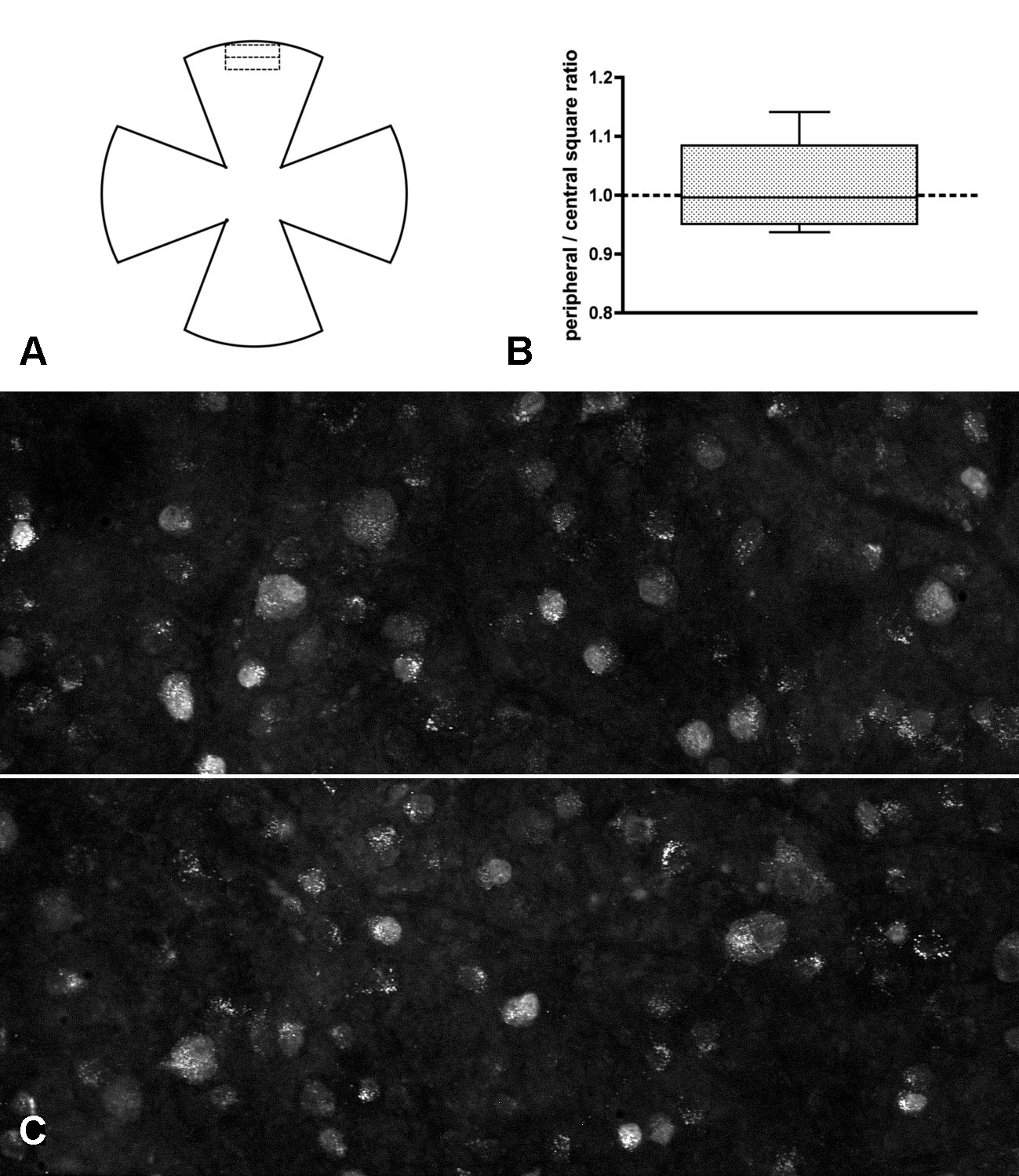

Supplement: Figure S1 — Quantification of RGC density changes in the outer periphery of the retina. A) Schematic of a flatmounted retina and the counting frame (dashed line), which is divided into a central and peripheral sub-frame. The size of the counting frame was enlarged for better visibility. B) Box plot of the peripheral to central subframe RGC density (p/c ratio) of 4 retinae. The dashed line indicates a p/c ratio of 1 (no difference between central and peripheral sub-frame). There is no statistically significant difference to the value 1 (p = 0.56) indicating that RGC density does not change as a function of eccentricity in the outer periphery. C) Representative image of FG labelled RGCs. Image size equals counting frame size. The horizontal white line indicates the boundary of the sub-frames. Top peripheral part, bottom central part. (TIF) [file pone.0038820.s003.tif]
